# The Effect of Botulinum Toxin Injections on Gross Motor Function for Lower Limb Spasticity in Children with Cerebral Palsy

**DOI:** 10.3390/toxins11110651

**Published:** 2019-11-08

**Authors:** Ja Young Choi, Seung Ki Kim, Eun Sook Park

**Affiliations:** 1 Department of Rehabilitation Medicine, Severance Hospital, Research Institute of Rehabilitation Medicine, Yonsei University College of Medicine, 50 Yonsei-ro, Seodaemun-gu, Seoul 03722, Korea; jaychoi3399@gmail.com (J.Y.C.); gsg1230@yuhs.ac (S.K.K.); 2 Department of Rehabilitation Medicine, Daejeon-Chungcheong Regional Rehabilitation Center, Chungnam National University Hospital, Chungnam National University College of Medicine, 266 Munhwa-ro, Jung-gu, Daejeon 35015, Korea

**Keywords:** cerebral palsy, spasticity, botulinum toxin, motor function

## Abstract

The aim of this study was to investigate the use of botulinum toxin type A (BoNT-A) injections and their efficacy on gross motor function for lower limb spasticity in children with spastic cerebral palsy (CP). This retrospective study included 919 injection occasions from 591 children with CP who received a lower limb BoNT-A injection between 2006 and 2016. The Gross Motor Function Measure (GMFM-88), the Modified Ashworth Scale, and the Modified Tardieu Scale were administered before and after injections. Injections were predominantly administered to children under the age of 6 years. The most common muscle injection site was the calf muscle for dynamic foot deformity. The second most commonly injected muscle was the hip adductor among 2–3 year olds and the hamstring muscle among 4–6 year olds. Distal injections were predominantly administered to high-functioning children, whereas proximal injections were typically administered to low-functioning children. Multilevel injections were mostly administered to midfunctioning children. GMFM-88 scores significantly increased post-injection for both high- and low-functioning groups. Younger age at injection and distal injection type were associated with larger improvements on the GMFM-88 at both short- and midterm follow-up. The target muscles for injection varied depending on gross motor functioning and age. Younger age at injection and distal injection type were significantly related with greater gain in gross motor function.

## 1. Introduction

Botulinum toxin type A (BoNT-A) injections are widely used to control lower limb spasticity in children with spastic cerebral palsy (CP) [1,2]. According to a population-based study, spasticity of the gastrocnemius muscle, as measured using the Modified Ashworth Scale (MAS), increases in most children with CP up to the age of 5, followed by a decrease up to the age of 15 [3]. In addition, a retrospective cohort registry study found that BoNT-A injections for children with CP differed according to age and level on the Gross Motor Function Classification System (GMFCS) [4]. 

During physical development in childhood, the contribution of non-neural components to hypertonia increases and leads to joint contracture or deformity. As for equinus of the foot, the increasing muscle stiffness and joint tightness leads to limitation of joint motion (LOM) as the child grows. Cast applications are known to be useful to improve the passive range of motion (ROM) at the ankle [5]. Therefore, according to an international consensus statement, cast application is recommended in combination with BoNT-A injections in the case of LOM at the ankle [6].

Although numerous previous studies have established that BoNT-A injections are effective in spasticity control and improving ROM [6], its efficacy in functional improvement is less certain [7]. In addition, the contributing factors involved in functional gain are still emerging.

Identification of related factors that allow for the prediction of the effect of BoNT-A injections could help to guide indications and treatment goals for the injection. Therefore, the aim of the present study was to investigate whether the efficacy of BoNT-A injections, or a combined case application and the targeting of different muscles, differed according to age and gross motor function. Additionally, the study aimed to elucidate whether BoNT-A injections could lead to functional gain and identify contributing factors relating to functional gain in children with spastic CP.

## 2. Results

### 2.1. Demographic Data

In total, 919 injection occasions from 591 participants were analyzed in this study. The sample included 340 boys and 251 girls, with a mean age at first injection of 4.68 years old (SD 2.22 years, age range 2–13 years). Among the participants, children classified as ambulatory CP comprised 73.6% of the sample (GMFCS level I/II/III: 210/111/114), and nonambulatory CP comprised 26.4% (GMFCS level IV/V: 91/65). Meanwhile, 454 participants (76.9%) had bilateral involvement, and 137 participants (23.1%) had unilateral involvement. The characteristics of the participants included in this study are described in Table 1. 

### 2.2. Injection Profile 

Injections were administered unilaterally on 284 occasions (30.9%) and bilaterally on 635 occasions (69.1%). Distal injections for foot deformity were given on 502 (54.6%) occasions, while proximal injections into the muscles of the hip and/or knee joints were administered on 80 (8.7%) occasions. Multilevel injections targeting the muscles of three joints—the hip, ankle, and/or knee—were given on 337 (36.7%) occasions (Table 2). The average injection dose of BoTN-A was 9.39 ± 4.82 units. The injection dose was significantly different depending on the type of injection (*p* < 0.05). 

The injection type was significantly different according to GMFCS level (Table 3). Participants with good functioning according to their GMFCS level were more likely to receive a distal, rather than proximal, injection. The distal injection group predominantly comprised children at a good level of functioning on the GMFCS, while the proximal injection group mainly encompassed children at a poor level of functioning on the GMFCS. In the multilevel injection group, the GMFCS level was widely distributed from GMFCS levels I to V, though participants at the midfunctioning level (GMFCS levels II to IV) made up the majority of this group (Table 3).

Injections were administered 1–11 times. However, most of the participants for this study received the injection within 3 times. Injection frequencies of 4 or more occurred on 150 occasions (16.3%; Table 4).

**Figure 1:**
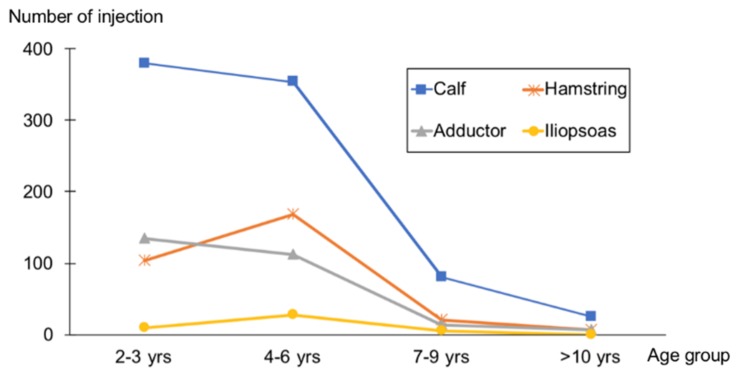
Target muscles according to age group. Calf refers to the gastrocnemius muscle with or without the soleus, tibialis posterior, or peroneus muscles. Hip adductor refers to the adductor longus muscle with or without the gracilis muscles. Hamstring refers to the semimembranosus and semitendinosus muscles.

The most commonly injected muscle in all age groups was the calf muscle to target dynamic foot deformity. The second most commonly injected muscle was the hip adductor in children aged 2–3 years and the hamstring muscle in children aged 4–6 years. Injections were predominantly administered to children under the age of 6 years (Figure 1). 

### 2.3. Reduction of Tone 

Significant improvements on the MAS and R1 (the angle of muscle reaction) on the MTS were observed for most of the muscles injected at the first and second follow-ups compared to baseline, but the tone in some of the muscles significantly increased at the second follow-up compared to the first follow-up (Table 5). As for passive ROM (R2 on the MTS), ankle dorsiflexion, hip abduction, and popliteal angles had significantly improved at the first follow-up, and these effects were maintained at the second follow-up.

### 2.4. Combined Short Leg Cast Application 

The frequency of cast application after injection was significantly different according to age group. The frequency of cast application was significantly higher in children aged over 4 years old compared to children aged 2–3 years (Table 6).

### 2.5. GMFM-88 Change

Both ambulatory and nonambulatory groups demonstrated significant gains on the Gross Motor Function Measure (GMFM-88) at both short and midterm follow-ups compared to baseline assessment, but only ambulatory children had significantly improved on the GMFM-88 at the second follow-up assessment (within 3−6 months) compared to the first follow-up assessment (within 1−2 months) (Table 7). 

**Figure 2:**
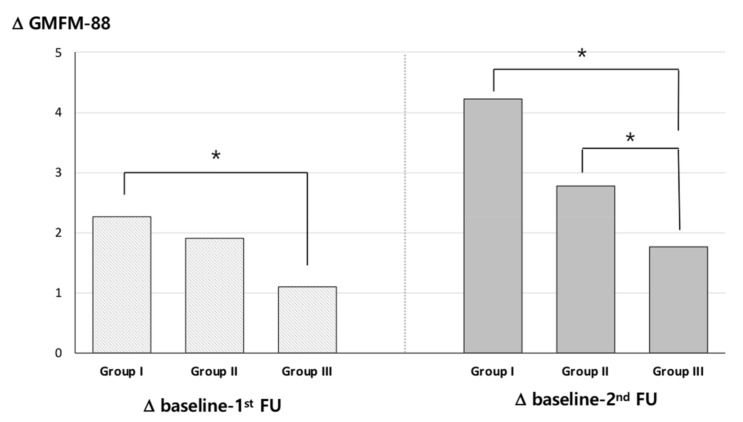
Changes in gross motor function according to multiple injection groups. Group I, first injection; Group II, second or third injection; Group III: multiple injections (≥4 times). * *p* < 0.05 using ANOVA with post hoc Bonferroni correction.

In addition, the present study revealed a significant improvement in GMFM-88 scores following multiple injections, but this improvement was significantly smaller compared to children who had received either one or two-to-three injections (Figure 2).

### 2.6. Factor Analysis of Changes in GMFM-88 Scores 

A univariate analysis of both short-term and midterm changes revealed that age at injection, injection type, and number of repeat injections were significant factors associated with gains on the GMFM-88. A multivariate analysis revealed that the age at injection and injection type continued to be significant factors associated with changes on the GMFM-88 at short- and midterm follow-up (Table 8).

## 3. Discussion

According to a previous population-based study, BoNT-A injections were predominantly administered to the calf muscle in ambulatory children (GMFCS level I to III) and to the hamstring and adductor muscles in nonambulatory children (GMFCS level IV to V) [4]. The literature indicates that multilevel injections were most commonly given to children at levels II to IV on the GMFCS, even though it could also be administered to children at levels I or V [8,9,10,11,12]. The results of our study are in line with these observations. Focal spasticity, such as equinus gait, is a more common issue than diffuse spasticity in children with good gross motor functioning. On the other hand, proximal muscle injections were commonly administered to children at level V on the GMFCS because of their high risk of hip dislocation or subluxation. Meanwhile, multilevel injections were typically given to children at levels II to IV on the GMFCS. These findings suggest that the target muscles for injection can differ depending on the level of gross motor functioning.

BoNT-A injections are known to be an effective intervention for dynamic spasticity but not static contracture. Therefore, children with spastic CP usually received the BoNT-A injection before the development of static contracture, which is often the target for orthopedic surgery. In a previous population-based study, BoNT-A injections were most frequently administered to children aged 4–6 years [4]. The results of the present study are in line with these previous findings. 

Equinus foot is the most common deformity in children with CP. A retrospective cohort registry study demonstrated that the gastrocnemius muscle was the most common muscle treated with BoNT-A injections irrespective of age or level of gross motor function [4]. In addition, the hip adductor muscle was the second most commonly targeted muscle among children under 4 years old, whereas the hamstring muscle was the second most commonly targeted muscle for BoNT-A injections among children over the age of 4, and reached a peak in children aged 10–12. The results of our study demonstrated a similar pattern. The degree of spasticity often decreases between 4–5 years and 12–15 years of age [3,13]. The combination of reduced muscle tone, increased body weight with age, and reduced muscle strength might contribute to the development of crouch gait. As such, hamstring muscle injections seem to be increasingly administered to older children. Compared to an earlier study conducted by Franzen et al. [4], the present study revealed an earlier peak, at the age of 4–6 years, for hamstring injections. This might be due to the fact that orthopedic intervention for the correction of musculoskeletal malalignment, such as joint contracture and dislocation, is commonly implemented from the age of seven at our institute. Taken together, these findings suggest that the use of BoNT-A injections as a therapeutic intervention, and the muscle group targeted varied depending on age.

Combined therapy—using a serial or single short leg cast with BoNT-A injections—is thought to achieve optimal improvements for equinus [14]. In our clinic, a cast was applied for participants with ankle joint LOM if there was no longstanding contracture. In children with spastic CP, the neural factor—increased muscle activity caused by velocity-dependent pathological stretch reflex by definition—is the main reason for increased resistance to passive joint motion. As a child grows, the non-neural mechanical properties of soft tissue and joint structures, such as stiffness and viscosity, can increasingly contribute to increased resistance to passive stretch. In a previous study, the range of dorsiflexion of the ankle joint decreased as the child grew to 18 years of age, while the annual decrease was fastest up to approximately 5–6 years of age [15]. Because of the increased contribution of non-neural factors for equinus foot as the child grows, cast application after injection was significantly more common in children aged over four years, compared to younger children. 

In previous studies, BoNT-A injections, or combined therapy with BoNT-A and physiotherapy, led to greater improvements on the GMFM-88 in children with CP than physiotherapy [8,16,17,18,19], although one report found conflicting evidence [20]. In addition, injection technique [21], gross motor function, and age [4,22] were suggested as factors associated with changes on the GMFM-88 [23]. 

Using BoNT-A injections as an intervention has a long-term effect on motor function, even though the effect of the injection on muscle tone had disappeared [9,24,25,26]. The degree of improvement in gross motor function with intensive physical therapy and repeated BoNT-A injections might be greater at long-term follow-up as the child develops and grows. In this context, a long-term follow-up study could lead to a greater gain in gross motor function after injection in combination with intensive physical therapy [8]. Therefore, the baseline was set as the GMFM-88 score before the last BoNT injection, but not the first injection, to minimize the influence of child development. In the present study, participants demonstrated some improvement on the GMFM-88 when they received four or more repeated injections, though the degree of improvement was smaller compared to the improvements seen after the first, and second or third injections. These findings are in line with the results of previous studies [24,27], in which the efficacy of BoNT-A apparently declined with repeated injections, with most children benefitting from two to three injections. Scores on the GMFM-88 reached a plateau between ages three and seven depending on their GMFCS level [28], and thus age has been reported as a significant factor associated with gains on the GMFM-88 after BoNT-A injection in previous studies [22,29] as well as the present study. 

The efficacy of BoNT-A injections has been proven in the treatment of spastic equinus in ambulant children with CP (GMFCS levels I to III) with the aim of improving gait pattern [30,31]. There are some positive reports showing gains on the GMFM-88 after administering injections to children with CP [8,9,22,24]. However, the question of whether BoNT-A injections in combination with physical therapy facilitates greater improvements in gross motor function is inconclusive because of the poor level of existing evidence, according to systematic reviews [23,32]. 

The effect of gross motor functioning in relation to functional gain, as measured by the GMFM-88, has rarely been reported. In a previous study, moderately impaired children (GMFCS level III) showed a tendency to attain higher gains in GMFM-88 scores, but these improvements were not statistically significant [22]. Another report showed that children with moderate functional impairment (GMFCS levels III to IV) demonstrated higher gains on the GMFM-88, but only one in five children at GMFCS level V showed improvements on the GMFM-88 [33]. In general, the use of BoNT-A injections in children with severe functional impairment is usually aimed at reducing pain, preventing hip dislocation, and improving ease in care and positioning [32]. Our study demonstrated significant gains in nonambulatory children. The efficacy of BoNT-A injections in terms of functional change on the GMFM-88 according to GMFCS level requires further investigation. Furthermore, it is of interest that injection type, not ambulatory function, was a factor associated with gains on the GMFM-88 in the present study. We observed that injection type differed based on the level of gross motor functioning on the GMFCS. The main purpose of BoNT-A injections for children at GMFCS level V was the prevention of hip subluxation and to ease care, thus proximal injections were predominantly performed. As such, injection type was significantly associated with changes on the GMFCS in the present study. To the best of our knowledge, there have been no reports examining functional gains according to injection type. Further studies of rigorous methodological quality are required. 

This was a retrospective study in a single center, though most of the included children with CP visited our clinic especially for BoNT-A injections. We established the protocol for children with CP who had received BoNT-A injections for the assessment, but some children were missed during the courses. However, this did not appear to significantly impact our results. In addition, the amount of physical therapy received after injection varied to some extent, but we did not expect the outcome to be significantly affected. As the importance of intensive physical therapy after injection was emphasized to the parents of children who had received BoNT-A injections, physical therapy was performed on most of the children. Regardless of these limitations, a strength of this study is that GMFM-88 changes could be assessed in a relatively large number of cases, enabling us to delineate factors associated with changes on the GMFM-88 after injection. 

## 4. Conclusions 

Our study revealed that BoNT-A injections are common under the age of six, and combined cast application after injection for equinus foot became increasingly more common from the age of four. The target muscles for injection varied depending on the level of gross motor function and age. The gross motor functional changes, as measured with the GMFM-88, were significantly associated with age at injection as well as injection type in children with spastic CP. 

## 5. Materials and Methods 

### 5.1. Study Design

This study was conducted in a pediatric spasticity management clinic in a university-affiliated, tertiary-care teaching hospital. In this clinic, children with CP were regularly followed by a multidisciplinary clinic team of three pediatric physiatrists, nurses, physiotherapists, and an occupational therapist.

For this study, the medical records of children with spastic CP who had received BoNT-A injections, with or without neurolysis or motor point block, with ethyl alcohol in the lower limb between January 2006 and December 2016 were retrospectively reviewed. The medical records of 2329 injection occasions of 1213 children were reviewed. Of these records, children under 13 with full assessments before injection and a follow up assessment at least once after injection were selected for this study. As a result, the data from a total of 919 injections from 591 children with CP were analyzed.

This study was approved by the Institutional Review Board of the Severance Hospital (4-2019-0587). Informed consent for BoNT-A injection was obtained from the parents of all children in this study. In addition, oral or written assent was also obtained from the children over 7 years old according to their understanding and cognitive abilities.

### 5.2. Intervention

At our clinic, children with spastic CP are regularly followed in order to provide appropriate interventions, including BoNT-A injections, in a timely manner. At every visit, spasticity, ROM, gross motor skills, and movement or gait patterns are assessed. In the case of patients receiving BoNT-A injections, the selection of muscles and the dosage to be injected was determined by the physician based on clinical assessments including the MAS, MTS, the Hypertonia Assessment Tool (HAT) if possible, and the GMFM-88. One vial containing 100 units of onabotulinum toxin-A (Botox®, Allergan Inc, Irvine, CA, USA) was diluted with 2 mL normal saline; 200 units of Neu-BoNT/A (Neuronox®, Medytox Inc, Ochang-eup, Cheongwon-gu, Cheongju-si, Chungcheongbuk-do, Republic of Korea) was diluted with 4 mL normal saline. For the children who received multilevel injections, nerve block with 50% ethyl alcohol was added if the total dose per one session exceeded the maximal safe dosage (a total of 16–20 U/kg of body weight depending on general health status, such as respiratory health or malnutrition, etc.). Obturator nerve block, tibial nerve block to the gastrocnemius muscle, or motor point block to the hamstring muscle was administered depending on whether additional nerve or motor point block was needed. Depending on the size and the severity of spasticity of the target muscles, 1–5 U/kg botulinum toxin was injected per muscle. The total muscle dose was divided between two to four injection sites depending on muscle size. Injections were guided by ultrasonography or by electrical stimulation for the exact identification of target muscles or nerve or motor point blocks. Electrical stimulation for nerve or motor point blocks was administered under general anesthesia. Distal injections were defined as those that were confined to the calf muscle when used to treat a foot deformity, such as equinus with or without varus or valgus foot. For distal injections, the gastrocnemius muscle was the main target muscle on all occasions. Meanwhile, the peroneus muscle or tibialis posterior muscle were also injected on nine occasions when equinus was accompanied by varus or valgus foot. In some hemiplegic children with equinus foot, both the soleus muscle and the gastrocnemius muscle were injected. Proximal injections were defined as those that were confined to the hip muscles, such as the hip adductor or iliopsoas muscle, and/or the hamstring muscle, without the calf muscle injection for foot deformity. Most of the children who had proximal injections had received the injection into both the hip adductor and/or hamstring muscles. Multilevel injections were defined as those that were distributed diffusely from hip to ankle joint muscles. For multilevel injections, the gastrocnemius, hamstring, hip adductor, iliopsoas, and rectus femoris were the target muscles based on the clinical assessment.

For children who had LOM at the ankle joint, a short leg cast was applied for one to three weeks (depending on the degree of LOM) to increase the ROM after injection. The short leg casts were applied with the patient in a prone position with the knee flexed to 90° and the ankle dorsiflexed to maximal attainable dorsiflexion and held in the neutral hind foot position. The consecutive cast was applied with a progressively increasing degree of ankle dorsiflexion to stretch the calf. The importance of intensive physical therapy after injection was emphasized to all parents. Subsequently, most of the children in the study received intensive physical therapy. 

### 5.3. Assessment 

Using the GMFM-88, a physical therapist assessed the gross motor function of children who received a BoNT-A injection in the lower limb. The physiatrist assessed muscle tone using the MAS and MTS by assessing the ROM in each joint and by determining the child’s GMFCS level. All children who received the injection were assessed before and after the injection was administered. Follow-up assessments were conducted 1–2 months post-injection, and again 3–6 months post-injection. 

The level of gross motor functioning of children with CP can be classified according to the GMFCS, which consists of five levels, from level I (most able) to level V (least able). The GMFCS is the most widely used system to classify gross motor function in children with CP. The GMFCS levels used for this study were determined based on classification at the last visit. High functioning was defined as ambulatory children at GMFCS level I to III, while low functioning was defined as nonambulatory children at GMFCS levels IV to V. 

Functional gross motor gains were assessed by physical therapists using the GMFM-88. Evidence indicates that the GMFM-88 is a valid and reliable measurement tool [34]. The short-term follow-up assessment was conducted between 1–2 months after injection, whereas the midterm follow-up assessment was conducted between 3–6 months after injection. For children who received multiple injections, the baseline assessment before the last injection was chosen for measuring the changes. 

### 5.4. Statistical Analyses

Statistical analysis was performed using the Statistical Package for the Social Sciences for Windows (SPSS version 20.0, IBM SPSS Incorporated, Chicago, IL, USA). Chi-squared tests were used to compare the distribution of injection types according to GMFCS level. Linear mixed or generalized estimating equation models were used to analyze the changes in GMFM-88 score, MTS angle, and MAS grade after intervention in each group. Post hoc Bonferroni correction was used for multiple comparisons. The independent *t* test was used to compare the differences in improvement between the groups. Additionally, univariate and multivariate linear regression modeling were used to identify factors significantly associated with functional change after injection. A *p*-value less than 0.05 was considered statistically significant for all statistical tests. 

## Figures and Tables

**Table 1:** Participants’ demographic and clinical features.

Characteristic	Total 591 Patients
Gender, male	340 (57.5%)
Topography classification	
Unilateral	137 (23.2%)
Bilateral	454 (76.8%)
GMFCS level (initial)	
I	210 (35.5%)
II	111 (18.8%)
III	114 (19.3%)
IV	91 (15.4%)
V	65 (11.0%)
Age at each injection (years)	4.68 ± 2.22 (2–13)

Values are expressed as mean ± SD (range) or number of participants (percentage); GMFCS, Gross Motor Functional Classification System.

**Table 2:** Injection profile characteristics.

Characteristic	919 Injections/591 Patients
**Types of injection**	
Distal injection only	502 (54.6%)
Proximal injection only	80 (8.7%)
Multi-level injection	337 (36.7%)
**Injection dose (unit)**	
Distal injection only	7.02 ± 3.19
Proximal injection only	9.84 ± 5.11
Multi-level injection	12.88 ± 4.65
**Injection site**	
Unilateral injection	284 (30.9%)
Bilateral injection	635 (69.1%)
**GMFM-88 assessment at each time interval**	
Baseline	919
1st follow-up (within 1–2 months after injection)	825
2nd follow-up (within 3–6 months after injection)	523

Values are expressed as mean ± SD (range) or number of participants (percentage); GMFM, Gross Motor Function Measure.

**Table 3:** Distribution of injection type according to GMFCS level.

GMFCS	Injection Site			*p*-Value	
	Distal	Proximal	Multilevel	Overall	Post-Hoc
**I**	299 (59.6%)	2 (2.5%)	49 (14.5%)	<0.001 *	Distal vs. proximal <0.001 * Distal vs. multilevel <0.001 * Proximal vs. multilevel = 0.329
**II**	102 (20.3%)	5 (6.3%)	73 (21.7%)		
**III**	46 (9.2%)	18 (22.5%)	100 (29.7%)		
**IV**	33 (6.6%)	20 (25.0%)	79 (23.4%)		
**V**	22 (4.4%)	35 (43.8%)	36 (10.7%)		

Values are number of cases (%); Chi-squared test with post hoc Bonferroni correction; GMFCS, Gross Motor Functional Classification System.

**Table 4:** Distribution of repeat injections.

Group	Injection Occasion	Injection Frequency	Total
I: 1st injection	1st	388	388 (42.2%)
II: 2nd–3rd injection	2nd	248	381 (41.5%)
	3rd	133	
III: Multiple injections (≥4 times)	4th	63	150 (16.3%)
	5th	40	
	6th	23	
	7th	12	
	8th	6	
	9th	4	
	10th	1	
	11th	1	

**Table 5:** Changes in tone in major muscles.

Injection Site	Assessed Muscle and Posture	Assessment Time			*p-*Value
		Baseline	1st Follow-Up	2nd Follow-Up	
**Modified Ashworth Scale, median (IQR)**					
Calf	Ankle with knee flexion	2 (1+, 2)	1+ * (1, 1+)	1+ * (1, 2)	<0.001 ^§^
Calf	Ankle with knee extension	2 (2, 3)	1+ * (1+, 2)	1+ *^,†^ (1+, 2)	<0.001 ^§^
Hamstring	Knee flexor	2 (1+, 2)	1+ * (1, 1+)	1+ * (1+, 2)	<0.001 ^§^
Hip adductor	Hip adductor with knee flexion	1+ (1+, 2)	1 * (1, 1+)	1+ *^,†^ (1, 2)	<0.001 ^§^
Hip adductor	Hip adductor with knee extension	2 (1+, 2)	1+ * (1, 1+)	1+ *^,†^ (1, 2)	<0.001 ^§^
**Modified Tardieu Scale, R1 (degree)**					
Calf	Ankle with knee flexion	−7.79 (15.09)	2.56 * (10.60)	−0.04 *^,†^ (13.49)	<0.001 ^§§^
Calf	Ankle with knee extension	−17.88 (14.28)	−4.90 * (13.38)	−10.80 *^,†^ (13.05)	<0.001 ^§§^
Hamstring	Knee flexor	67.24 (17.11)	49.22 * (16.18)	59.69 *^,†^ (21.25)	<0.001 ^§§^
Hip adductor	Hip adductor with knee flexion	35.71 (13.84)	48.80 * (12.90)	43.08 * (14.98)	<0.001 ^§§^
Hip adductor	Hip adductor with knee extension	16.93 (7.85)	28.75 * (8.33)	23.72 *^,†^ (10.62)	<0.001 ^§§^
**Modified Tardieu Scale, R2 (degree)**					
Calf	Ankle with knee flexion	17.14 (12.73)	23.36 * (11.33)	21.93 * (11.50)	<0.001 ^§§^
Calf	Ankle with knee extension	7.05 (11.72)	13.32 * (10.73)	11.34 * (10.77)	<0.001 ^§§^
Hamstring	Knee flexor	38.49 (15.65)	29.79 * (12.92)	36.35 * (16.04)	<0.001 ^§§^
Hip adductor	Hip adductor with knee flexion	57.95 (12.13)	63.21 * (10.48)	61.63 (13.93)	0.001 ^§§^
Hip adductor	Hip adductor with knee extension	30.96 (10.19)	38.56 * (7.26)	34.63 * (9.63)	<0.001 ^§§^

Values are expressed as median (IQR) or mean (SD); 1st follow-up, within 1–2 months after injection; 2nd follow-up, within 3–26 months after injection; ^§^ *p* < 0.05, generalized estimating equations (GEE) model, ^§§^ *p* < 0.05, linear-mixed model; * post hoc analysis, compared with baseline, *p* < 0.05; ^†^ post hoc analysis, compared with 1st follow-up data, *p* < 0.05.

**Table 6:** Distribution of children treated with casts in different age groups.

Serial Cast	Age Group at Each Injection				*p*-Value	
	I: 2–3 Years	II: 4–6 Years	III: 7–9 Years	IV: >10 Years	Overall	Post-Hoc
**Cast/Total**	50/403 (12.4%)	83/393 (21.1%)	31/92 (33.7%)	12/31 (38.7%)	<0.001 *	I vs. II = 0.006 * I vs. III < 0.001 * I vs. IV < 0.001 * II vs. III = 0.084 II vs. IV = 0.246 III vs. IV > 0.999

* *p* < 0.05, Chi-square test with post-hoc Bonferroni correction.

**Table 7:** Changes in GMFM-88 score after injection.

	GMFM-88			*p*-Value
	Baseline	1^st^ Follow-Up	2^nd^ Follow-Up	
**Functional level**				
Ambulatory (GMFCS I-III)	76.57 (20.83)	78.77 (19.47) *	80.96 (18.70) *^,†^	<0.001^§^
Non-ambulatory (GMFCS IV-V)	30.27 (20.98)	32.00 (21.71) *	34.32 (19.78) *	<0.001^§^
**Injection occasion**				
1st injection	59.55 (30.30)	61.38 (29.97) *	64.95 (28.37) *^,†^	<0.001^§^
2nd-3rd injection	67.52 (27.54)	70.84 (27.96) *	70.37 (27.96) *^,†^	<0.001^§^
4th or more injection	74.17 (25.30)	76.20 (23.78) *	75.48 (25.27) *	0.005 ^§^

Values are expressed as mean (SD); 1st follow-up, within 1–2 months after injection; 2nd follow-up, within 3–6 months after injection; ^§^ *p* < 0.05, linear-mixed model; * post hoc analysis, compared with baseline, *p* < 0.05; ^†^ post hoc analysis, compared with 1st FU data, *p* < 0.05.

**Table 8:** Linear regression analysis of factors associated with GMFM-88 total change.

	At Primary End-Point (1–2 Months after Injection)		At Follow-Up (3–6 Months after Injection)	
	Univariate	Multivariate	Univariate	Multivariate
Variable	B (SE)	B (SE)	B (SE)	B (SE)
**Age at injection**	−0.30 * (0.07)	−0.23 * (0.07)	−0.63 * (0.13)	−0.51 * (0.15)
**GMFCS**		-		-
Level I-III	ref		ref	
Level IV-V	−0.42 (0.34)		−0.86 (0.69)	
**Body involvement**		-		-
Unilateral	ref		ref	
Bilateral	0.61 (0.36)		0.97 (0.18)	
**Injection type**				
Distal only	ref	ref	ref	ref
Proximal only	−1.46 * (0.54)	−1.31 * (0.54)	−3.08 * (1.10)	−2.75 * (1.07)
Multilevel	0.73 * (0.31)	0.60 (0.31)	0.41 (0.64)	0.11 (0.63)
**Injection Occasion**				
1st injection	ref	ref	ref	ref
2nd, 3rd injection	−0.35 (0.32)	−0.23 (0.32)	−1.46 * (0.66)	−0.99 (0.66)
≥4th injection	−1.16 * (0.45)	−6.51 (0.47)	−2.48 * (0.88)	−1.22 (0.93)

* *p* < 0.05 using linear regression analysis. SE, standard error; GMFCS, Gross Motor Functional Classification System; ref, reference group.
